# Fluoroquinolone-Resistant and Extended-Spectrum β-Lactamase–Producing *Escherichia coli* Infections in Patients with Pyelonephritis, United States^1^

**DOI:** 10.3201/eid2209.160148

**Published:** 2016-09

**Authors:** David A. Talan, Sukhjit S. Takhar, Anusha Krishnadasan, Fredrick M. Abrahamian, William R. Mower, Gregory J. Moran, EMERGEncy ID Net Study Group

**Affiliations:** David Geffen School of Medicine at the University of California, Los Angeles (UCLA), Los Angeles, California, USA (D.A. Talan, A. Krishnadasan, F.M. Abrahamian, W.R. Mower, G.J. Moran);; Olive View–UCLA Medical Center, Los Angeles (D.A. Talan, A. Krishnadasan, F.M. Abrahamian, G.J. Moran);; Brigham and Women’s Hospital, Boston, Massachusetts, USA (S.S. Takhar);; Harvard Medical School, Boston (S.S. Takhar);; Ronald Reagan Medical Center, Los Angeles (W.R. Mower)

**Keywords:** *Escherichia coli*, *E. coli*, pyelonephritis, prevalence, risk factors, fluoroquinolone, extended-spectrum β-lactamase, ESBL, *Enterobacteriaceae*, antimicrobial resistance, CTX-M-15, complicated, uncomplicated, bacteria, gram-negative bacteria, nephritis, kidney disease, United States

## Abstract

For 2013–2014, we prospectively identified US adults with flank pain, temperature >38.0°C, and a diagnosis of acute pyelonephritis, confirmed by culture. Cultures from 453 (86.9%) of 521 patients grew *Escherichia coli.* Among *E. coli* isolates from 272 patients with uncomplicated pyelonephritis and 181 with complicated pyelonephritis, prevalence of fluoroquinolone resistance across study sites was 6.3% (range by site 0.0%–23.1%) and 19.9% (0.0%–50.0%), respectively; prevalence of extended-spectrum β-lactamase (ESBL) production was 2.6% (0.0%–8.3%) and 12.2% (0.0%–17.2%), respectively. Ten (34.5%) of 29 patients with ESBL infection reported no exposure to antimicrobial drugs, healthcare, or travel. Of the 29 patients with ESBL infection and 53 with fluoroquinolone-resistant infection, 22 (75.9%) and 24 (45.3%), respectively, were initially treated with in vitro inactive antimicrobial drugs. Prevalence of fluoroquinolone resistance exceeds treatment guideline thresholds for alternative antimicrobial drug strategies, and community-acquired ESBL-producing *E. coli* infection has emerged in some US communities.

## Abstract 2

Prevalence of fluoroquinolone resistance now exceeds treatment guideline thresholds for alternative antimicrobial drug strategies.

*Escherichia coli,* the predominant cause of community-acquired urinary tract infection (UTI) worldwide, is increasingly resistant to available antimicrobial drugs. In the United States, in vitro resistance of *E. coli* to trimethoprim/sulfamethoxazole (TMP/SMX) became prevalent in the 1990s (*1*). Over the past decade, fluoroquinolone resistance rates have increased to >10% in some surveys (*2**,**3*).

In many parts of the world, *E. coli* fluoroquinolone resistance rates are >20% among patients with community-acquired uncomplicated UTI and >50% among patients with complicated infections (*4*). In addition, infections resulting from extended-spectrum β-lactamase (ESBL)–producing *E. coli* and other *Enterobacteriaceae* are becoming increasingly common in these same areas and are associated with sequence type (ST) 131, a globally disseminated, multidrug-resistant clone that frequently produces CTX-M-15 ESBL. These *E. coli* isolates are generally resistant to cephalosporins and often to other antimicrobial drug classes. In North America, ESBL-producing *E. coli* infections have occurred predominantly in patients with healthcare exposure and have not become prevalent as a cause of community-acquired infections (*5**–**8*).

The 2010 international treatment guidelines of the Infectious Disease Society of America (IDSA) recommend for acute uncomplicated pyelonephritis a fluoroquinolone and an initial dose of an agent from another antimicrobial drug class (e.g., ceftriaxone or gentamicin) if the fluoroquinolone resistance rate is >10% (*9*). For uncomplicated cystitis, the guidelines discourage use of an antimicrobial drug if its resistance rate is >20%. The guidelines do not address a scenario in which ESBL-producing uropathogens have become prevalent among patients with community-acquired infections. Use of antimicrobial drugs for which the uropathogen shows in vitro resistance has been associated with substantially reduced response rates (*1**,**10**,**11*), which can lead to serious consequences, particularly for patients with pyelonephritis. Given rapid changes in global resistance patterns and a lack of recent active and prospective surveillance of community-acquired UTI in the United States, the extent to which the prevalence of fluoroquinolone resistance has increased and multidrug-resistant ESBL-producing strains have emerged in the community is unknown.

We sought to determine the prevalence of *E. coli* antimicrobial resistance among patients with acute pyelonephritis who sought care at a US emergency department (ED)–based sentinel research network. We focused on fluoroquinolone-resistant and ESBL-producing isolates from these patients and examined risk factors for antimicrobial drug resistance.

## Methods

### Participants

We recruited adults seeking care in *EMERGE*ncy ID NET, a network of 10 university-affiliated urban US EDs (*12*). All 10 study sites (Technical Appendix) provided institutional review board approval. 

We enrolled patients >18 years of age who sought care during July 2013–December 2014 and had flank pain or costovertebral tenderness; temperature >38.0°C (100.4°F) measured by any method (i.e., oral, rectal, or axillary); and a presumptive diagnosis of acute pyelonephritis (i.e., patient received treatment for this infection during ED visit or was prescribed treatment at discharge). All sites conducted an audit to compare characteristics of enrolled and nonenrolled eligible patients to estimate case-finding sensitivity and detect enrollment biases (online Technical Appendix). 

### Design and Measurements

We conducted a cross-sectional study by using a convenience sample of prospectively identified patients. ED physicians or study coordinators who used standardized forms at the time of care collected the following: demographic characteristics (i.e., age, sex, race, ethnicity); symptom duration; urinary tract abnormalities; UTI within the previous year; concurrent and immunocompromising conditions; antimicrobial drug use within the previous 2 and 60 days; a fluoroquinolone- or ceftriaxone-resistant UTI within the previous 90 days and 1 year; long-term care residence; hospitalization; travel outside North America within the previous 90 days; illness severity; disposition; and treatments provided. The study population consisted of patients with urine specimens that grew a single uropathogen at >10^4^ CFU/mL. We defined a uropathogen as an organism known to be associated with UTI; among possible pathogens, we found *E. coli*, *Klebsiella*, *Proteus*, *Pseudomonas*, *Citrobacter*, *Enterobacter*, and *Salmonella* species; *Staphylococcus aureus* or *S.* *saprophyticus*; and *Enterococcus* and *Aerococcus* species. If a urine specimen grew >1 organism, we considered it to be contaminated and excluded it. We also excluded specimens that grew *Lactobacillus*, non-*saprophyticus* coagulase-negative *Staphylococcus*, or *Corynebacteria* species; or α- or γ-hemolytic streptococci. 

Patients’ urine specimens were collected in sterile containers by mid-stream clean-catch technique (91.4%), urethral catheterization (5.8%), and other techniques (e.g., sample from collection bag [2.7%] or suprapubic aspirate [0.2%]). Laboratories determined MICs by using automated susceptibility testing with VITEK commercial panels (bioMérieux, Marcy l’Etoile, France) at 7 sites; Microscan (Dade Behring, Inc., Sacramento, CA, USA) at 2 sites; and Phoenix Instrument System (Becton Dickinson, Franklin Lakes, NJ, USA) at 1 site, according to manufacturer instructions.

Each site used *E. coli* antimicrobial drug resistance breakpoints based on MIC breakpoints (µg/mL) of the Clinical and Laboratory Standards Institute (Wayne, PA, USA) as follows: ampicillin >32; TMP/SMX >4/76; cefazolin >8; ceftriaxone >4; cefotaxime >4; ciprofloxacin >4; levofloxacin >8; ertapenem >2; and imipenem >4 (*13*). We report resistance rates for antimicrobial drugs included in the study site laboratories’ standard susceptibility testing panel. In 2010, the Clinical and Laboratory Standards Institute changed breakpoints for cephalosporins, aztreonam, and carbapenems. We provided Etests (bioMérieux) for ceftriaxone and imipenem to sites that had not yet updated automated susceptibility testing systems to enable these sites to use the new breakpoints. We considered *Enterobacteriaceae* isolates that were nonsusceptible to ceftriaxone (i.e., MIC >1 µg/mL) to be potentially ESBL producing (*13*). The sites shipped these isolates to a reference laboratory to confirm speciation, ESBL production, and molecular characterization (online Technical Appendix).

We classified patients as having complicated pyelonephritis if they were pregnant or male or had a current or preexisting functional or anatomic urinary tract abnormality or current immunocompromising condition. Possible preexisting urinary tract abnormalities were history of kidney stones, genitourinary procedures within the past 30 days, prostatic pathology, bladder catheter within the past 30 days, neurogenic urinary retention, ureteral stricture, duplicated collecting system, renal or bladder cancer, renal transplant, ureteral diversion, vesico-ureteral reflux, single kidney, and nephrostomy tubes. Immunocompromising conditions were diabetes, active cancer, systemic corticosteroid use, current use of other immunosuppressants, chronic debilitating condition, chronic renal insufficiency or failure, and HIV infection. We identified current complicating features on the basis of clinical findings or laboratory studies in the ED; possible complications were pregnancy, diabetes, bladder catheter, ureteral stent, percutaneous nephrostomy tube, prostatitis, nephrolithiasis, renal or perirenal abscess, and urinary retention. We recorded history of chronic debilitating illness, such as chronic obstructive pulmonary disease or heart or hepatic failure, but did not assign such illness as a criterion for complicated infection. We classified patients without criteria for complicated pyelonephritis as having uncomplicated pyelonephritis. We defined healthcare-associated infections as those in patients hospitalized or residing in a long-term care facility within the previous 90 days; other patients were classified as having community-acquired infections.

### Statistical Analysis

To manage the study data, we used REDCap electronic-data capture tools hosted by the University of California, Los Angeles, CA, USA (*14*). We used SAS Version 9.3 (Cary, NC, USA) and Microsoft Excel 2013 (Redmond, WA, USA) to analyze data and used descriptive statistics to summarize patient characteristics and resistance prevalence. We calculated relative risks and 95% CIs to determine associations between epidemiologic and clinical characteristics and presence or absence of fluoroquinolone-resistant and ESBL-producing *E. coli* infections.

## Results

Of 817 enrolled patients with acute pyelonephritis, 793 (97.1%) submitted a urine culture. Of those 793 patients, 272 (34.3%) were excluded from analysis: 149 (18.8%) had no culture growth; 74 (9.3%) grew >1 contaminant; 17 (2.1%) grew >1 isolate at <10^4^ CFU/mL; 25 (3.2%) grew >1 organism at 10^4^ CFU/mL; and 7 (0.9%) had no fever. The study population consisted of 521 patients who grew 1 uropathogen at >10^4^ CFU/mL. The case finding audit revealed a 66% enrollment of eligible patients. Enrolled and nonenrolled patients were similar for most characteristics, including *E. coli* susceptibility rates to TMP/SMX, ceftriaxone, and fluoroquinolones (Technical Appendix).

Among the 521 study patients, median age was 37 (range 18–88, interquartile range 26–52) years; 455 (87.3%) were female (Table 1). Most (446 [85.6%]) patients had a community-acquired infection; 74 (14.2%) had a healthcare-associated infection (70 with hospitalization and 9 with nursing home residence in the previous 90 days). A total of 286 (54.9%) patients had uncomplicated pyelonephritis; 235 (45.1%) had complicated pyelonephritis.

**Table 1:** Epidemiologic, clinical, and laboratory characteristics of 521 US emergency department patients with acute uncomplicated and complicated pyelonephritis, July 2013–December 2014*

	Value		
Total patients, N = 521	Uncomplicated, n = 286	Complicated, n = 235
Age, median y (IQR; range)	37 (26–52; 18–88)	30 (23–41; 18–79)	50 (36–58; 19–88)
Symptom duration, median d (IQR; range)	3.0 (2–5; 0–30)	3.0 (2–5; 0–30)	3.0 (2–5; 0–30)
Initial ED temperature, °C (IQR; range)	38.9 (38.4–39.4; 38.0–43.0)	38.9 (38.4–39.4; 38.0–40.3)	39.0 (38.4–39.4; 38.0–43.0)
Sex			
F	455 (87.3)	286 (100.0)	169 (71.9)
M	66 (12.7)	0 (0)	66 (28.1)
Race/ethnicity			
White/Hispanic	372 (71.4)	191 (66.8)	181 (77.0)
Black	119 (22.8)	76 (26.6)	43 (18.3)
Asian/Pacific Islander	22 (4.2)	15 (5.2)	7 (3.0)
Other	18 (3.5)	11 (3.9)	7 (3.0)
Unknown	5 (1.0)	3 (1.0)	2 (0.9)
Hispanic ethnicity			
Yes	281 (53.9)	155 (54.2)	126 (53.6)
No	233 (44.7)	126 (44.1)	107 (45.5)
Unknown	7 (1.3)	5 (1.7)	2 (0.9)
Antimicrobial drugs taken			
Within past 60 d	125 (24.0)	49 (17.1)	76 (32.3)
Within past 2 d	36 (6.9)	15 (5.2)	21 (8.9)
Healthcare-associated illness†	74 (14.2)	18 (6.3)	56 (23.8)
Complicating feature			
Concurrent condition	131 (25.1)	0 (0)	131 (55.7)
History of UTA	116 (22.3)	0 (0)	116 (49.4)
Current feature	61 (11.7)	0 (0)	61 (26.0)
UTIs within past year‡			
0	334 (64.5)	196 (68.8)	138 (59.2)
1	86 (16.6)	46 (16.1)	40 (17.2)
2	428 (8.1)	23 (8.1)	19 (8.2)
≥3	56 (10.8)	20 (7.0)	36 (15.5)
Travel outside North America within past 90 d§	17/520 (3.3)	8/286 (2.8)	9/234 (3.8)
Prior UTI caused by fluoroquinolone-resistant *E. coli*			
Within past year	16 (3.1)	4 (1.4)	12 (5.1)
Within past 90 d	14 (2.7)	2 (0.7)	12 (5.1)
Prior UTI caused by ceftriaxone-resistant *E. coli*			
Within past year	9 (1.7)	2 (0.7)	7 (3.0)
Within past 90 d	6 (1.2)	1 (0.3)	5 (2.1)
Severity of Illness¶			
Mild	66 (12.7)	34 (11.9)	32 (13.6)
Moderate	267 (51.2)	156 (54.5)	111 (47.2)
Severe	188 (36.1)	96 (33.6)	92 (39.1)
Disposition			
Ward	240 (46.1)	100 (35.0)	140 (59.6)
MCA	40 (7.7)	13 (4.5)	27 (11.5)
Home	239 (45.9)	172 (60.1)	67 (28.5)
AMA	2 (0.4)	1 (0.3)	1 (0.4)

*Values are given as no. (%) except as indicated. AMA, left against medical advice; *E. coli* , *Escherichia coli*; ED, emergency department; IQR, interquartile range; MCA, monitored care admission; UTA, urinary tract abnormality; UTI, urinary tract infection..  †Hospitalized or residing in a long-term care facility within past 90 days. ‡Percentages were calculated with UTI information available for 518 patients, 285 of whom had uncomplicated cases and 233 had complicated cases.  §Percentages were calculated with information available for 520 patients, 286 of whom had uncomplicated cases and 234 had complicated cases. ¶Mild indicates illness that does not affect patient’s normal activities; moderate partially affects normal activities but does not confine patient to house or bed; severe affects activities considerably, such as confining patient to house or bed.

Of types of uropathogens in patients with uncomplicated and complicated pyelonephritis (Table 2), *E. coli* accounted for infections in 453 (86.9%) patients; 272 (60.0%) of the infections were uncomplicated, and 181 (40.0%) were complicated. Among the 286 patients with uncomplicated infections, *E. coli* accounted for 95.1%; among the 235 patients with complicated infections, *E. coli* accounted for 77.0% (Table 2).

**Table 2:** Uropathogens identified among US emergency department patients with acute uncomplicated and complicated pyelonephritis, July 2013–December 2014

Uropathogen	No. (%)		
	Total, N = 521	Uncomplicated cases, n = 286	Complicated cases, n = 235
*Escherichia coli*	453 (86.9)	272 (95.1)	181 (77.0)
*Staphylococcus saprophyticus*	2 (0.4)	2 (0.7)	0 (0)
*Staphylococcus aureus*	4 (0.8)	0 (0)	4 (1.7)
*Proteus* sp.	4 (0.8)	3 (1.0)	1 (0.4)
*Enterobacter* sp.	5 (1.0)	1 (0.3)	4 (1.7)
*Klebsiella pneumoniae*	25 (4.8)	4 (1.4)	21 (8.9)
*Enterococcus* sp.	12 (2.3)	0 (0)	12 (5.1)
*Pseudomonas* sp.	7 (1.3)	0 (0)	7 (3.0)
Group B streptococcus	2 (0.4)	1 (0.3)	1 (0.4)
Other*	5 (1.0)	2 (0.7)	3 (1.3)

*Other pathogens were 2 *Aerococcus urinae*, 2 *Citrobacter koseri*, and 1 *Salmonella* species.

*E. coli* antimicrobial drug resistance rates among patients with complicated pyelonephritis tended to be higher than rates for patients with uncomplicated cases, except for TMP/SMX (Table 3). Among all patients, *E. coli* resistance rates varied by drug: ampicillin, 57.2% (259/453); TMP/SMX, 36.4% (165/453); gentamicin, 9.9% (43/436); cefazolin, 14.2% (52/367); ceftriaxone, 7.7% (35/453); levofloxacin, 10.2% (33/325); and ciprofloxacin, 12.1% (48/397). Among 53 fluoroquinolone-resistant isolates, 8 (15.1%) were susceptible to ampicillin, 34 (64.2%) to gentamicin, and 27 (50.9%) to ceftriaxone. Of 46 isolates tested for susceptibility to cefazolin, 19 (41.3%) were susceptible; all 48 (100%) isolates tested were susceptible to a carbapenem. *E. coli* antimicrobial drug resistance rates for patients with uncomplicated and complicated pyelonephritis varied by study site (Technical Appendix Tables 2, 3).

**Table 3:** Antimicrobial drug resistance rates for *Escherichia coli* isolates from US emergency department patients with acute uncomplicated and complicated pyelonephritis, July 2013–December 2014*

Antimicrobial drug	Patients with antimicrobial drug–resistant isolates, no./no. tested (%)		
	Total, N = 453	Uncomplicated cases, N = 272	Complicated cases, N = 181
Trimethoprim/sulfamethoxazole	165/453 (36.4)	111/272 (40.8)	54/181 (29.8)
Ampicillin	259/453 (57.2)	152/272 (55.9)	107/181 (59.1)
Cefazolin	52/367 (14.2)	18/219 (8.2)	34/148 (23.0)
Ceftriaxone	35/453 (7.7)	7/272 (2.6)	28/181 (15.5)
Ciprofloxacin	48/397 (12.1)	15/237 (6.3)	33/160 (20.6)
Levofloxacin	33/325 (10.2)	10/195 (5.1)	23/130 (17.7)
Gentamicin	43/436 (9.9)	19/261 (7.3)	24/175 (13.7)
Imipenem	0/135 (0)	0/90 (0)	0/45 (0)
Ertapenem	0/201 (0)	0/111 (0)	0/90 (0)
Meropenem	0/161 (0)	0/96 (0)	0/65 (0)
Doripenem	0/139 (0)	0/74 (0)	0/65 (0)

*Denominators indicate number of isolates tested against a specific antimicrobial drug; the composition of testing panels varied by site.

**Figure 1:**
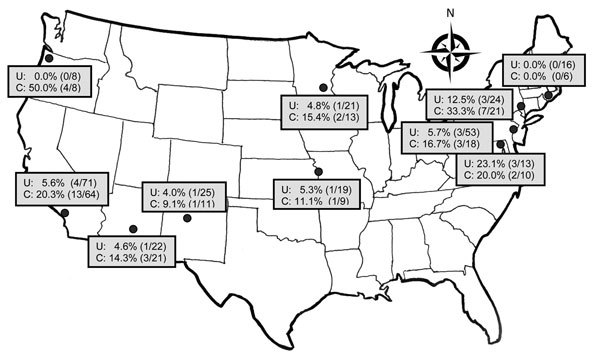
Prevalence of fluoroquinolone-resistant *Escherichia coli* infection among emergency department patients with uncomplicated (U) and complicated (C) pyelonephritis by study site, United States, July 2013–December 2014. Study sites are listed in the Technical Appendix; Technical Appendix Tables 2 and 3 provide additional results on antimicrobial resistance rates. In vitro resistance to ciprofloxacin and/or levofloxacin is shown as % (no. of patients with a resistant isolate/total no. of patients tested)

Among patients with uncomplicated pyelonephritis, 17 (6.3%) of 272 *E. coli* isolates were resistant to fluoroquinolone. The range of prevalence by site was 0.0%–23.1%; for 2 sites, prevalence was >10% (Figure 1; Technical Appendix Tables 2, 3). Among patients with complicated pyelonephritis, 36 (19.9%) of 181 *E. coli* isolates showed fluoroquinolone resistance. The range of prevalence by site was 0.0%–50.0%; 8 sites had a prevalence >10%, 4 of which had a prevalence >20%. We found fluoroquinolone resistance associated with complicated *E. coli* infection, prior use of antimicrobial drugs and fluoroquinolone, hospital admission, and prior UTI resulting from a fluoroquinolone- or ceftriaxone-resistant organism (Table 4).

**Table 4:** Factors associated with fluoroquinolone resistance among 453 US emergency department patients with pyelonephritis caused by *Escherichia coli,* July 2013–December 2014*

Factor	Fluoroquinolone-resistance rate		Relative risk (95% CI)
	Factor present, no./total (%)	Factor absent, no./total (%)	
Complicated infection	36/181 (19.9)	17/272 (6.3)	3.2 (1.8–5.8)
Prior antimicrobial drugs taken			
Within past 60 d	24/94 (25.5)	29/359 (8.1)	3.2 (1.9–5.3)
Within past 2 d	9/27 (33.3)	44/426 (10.3)	3.2 (1.6–5.8)
Prior fluoroquinolone use			
Within past 60 d	12/19 (63.2)	41/434 (9.4)	6.7 (3.8–9.6)
Within past 2 d	6/8 (75.0)	47/445 (10.6)	7.1 (3.2–9.4)
IV antimicrobial drugs within past 30 d	6/26 (23.1)	47/425 (11.1)	2.1 (0.8–4.3)
LTC within past 90 d	1/3 (33.3)	52/450 (11.6)	2.9 (0.2–7.8)
Admitted to hospital within 90 d	11/42 (26.2)	42/411 (10.2)	2.6 (1.3–4.6)
Travel outside United States within past 90 d	5/17 (29.4)†	48/436 (11.0)	2.7 (1.0–5.5)
UTI resulting from fluoroquinolone-resistant *E. coli*			
Within past year	7/9 (77.8)	46/444 (10.4)	7.5 (3.7–9.6)
Within past 90 d	7/8 (87.5)	46/445 (10.3)	8.5 (4.3–9.8)
UTI resulting from ceftriaxone-resistant *E. coli*			
Within past year	5/6 (83.3)	48/447 (10.7)	7.8 (3.3–9.4)
Within past 90 d	5/5 (100.0)	48/448 (10.7)	9.3 (4.1–9.3)

*Denominators differ because factors have a different distribution among the patient population. IV, intravenous; LTC, residence in a long-term care facility; UTI, urinary tract infection.  †Of the 5 patients that traveled outside the United States, 3 traveled to Mexico or Central America and 2 traveled to Asia.

**Figure 2:**
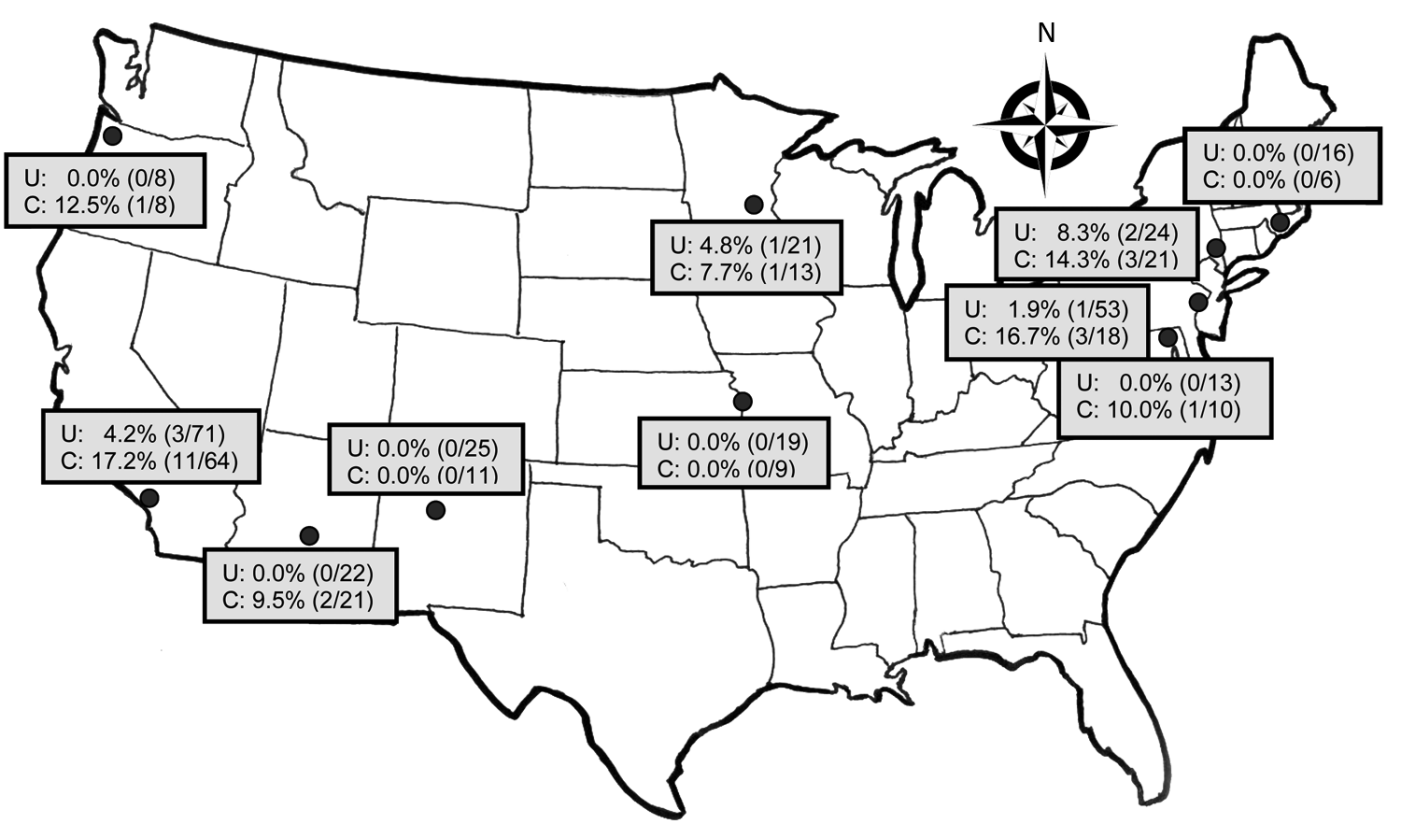
Prevalence of extended-spectrum β-lactamase–producing *Escherichia coli* infection among patients with uncomplicated (U) and complicated (C) pyelonephritis, by study site, United States, July 2013–December 2014. Study sites are listed in the Technical Appendix; Technical Appendix Tables 2 and 3 provide additional results on antimicrobial resistance rates.

**Figure 3:**
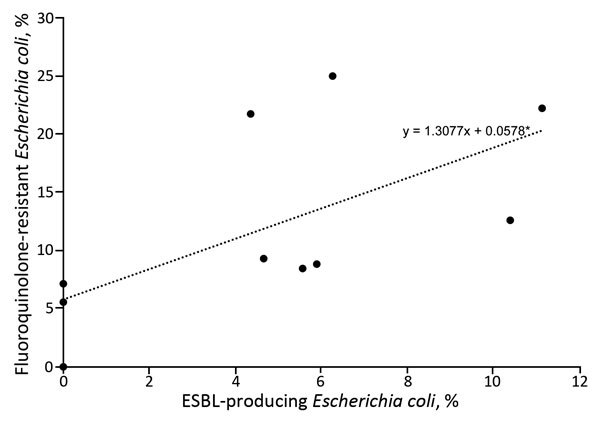
Prevalence of fluoroquinolone-resistant and ESBL-producing *Escherichia coli* infections among patients with uncomplicated and complicated pyelonephritis, by study site, United States, July 2013–December 2014. Each dot indicates a study site; the line to show the general trend between fluoroquinolone resistance and ESBL-producing *E. coli* was generated by using simple linear regression. ESBL, extended spectrum β-lactamase.

Among patients with uncomplicated pyelonephritis, ESBL production was found in 7 (2.6%) of 272 *E. coli* isolates; range by study site was 0.0%–8.3% (Figure 2; Technical Appendix Tables 2, 3). Among patients with complicated pyelonephritis, ESBL production was found in 22 (12.2%) of 181 *E. coli* isolates; range by site was 0.0%–17.2% (Figure 2; Technical Appendix Tables 2, 3). Frequencies of ESBL-producing E. coli isolates were higher among patients with antimicrobial drug resistance risk fac-tors than among those without these factors (Table 5). Nineteen (65.5%) of 29 patients with ESBL-producing *E. coli* infection had a recognized risk factor for antimicrobial drug resistance (Figure 2; Technical Appendix Tables 2, 3). Sixteen (55.2%) had antimicrobial drug exposure within the previous 60 days. During the previous 90 days, 6 (20.7%) had healthcare-setting exposure and 4 (13.8%) had travel outside North America. We found ESBL-producing *E. coli* infection associated with complicated infection, prior antimicrobial drug use, travel outside North America, and prior UTI resulting from a fluoroquinolone- or ceftriaxone-resistant organism. Among 37 isolates that grew other *Enterobacteriaceae*, including *Klebsiella pneumoniae*, 1 (2.7%) was ESBL producing. Among 29 ESBL-producing *E. coli* isolates, susceptibility rates to other antimicrobial drugs were 41.4% to TMP/SMX, 18.5% to ciprofloxacin, 21.7% to levofloxacin, 41.4% to gentamicin, and 100% to carbapenem. The prevalence of *E. coli* fluoroquinolone resistance correlated with the prevalence of ESBL-producing *E. coli* by site (Figure 3). 

**Table 5:** Factors associated with ESBL production among 453 US emergency department patients with pyelonephritis caused by *Escherichia coli,* July 2013–December 2014*

Factor	ESBL-producing *E. coli* rate		Relative risk (95% CI)
	Factor present, no./total (%)	Factor absent, no./total (%)	
Age >65 y	4/22 (18.2)	25/431 (5.8)	3.1 (0.96–8.1)
Complicated infection	22/181 (12.2)	7/272 (2.6)	4.7 (2.0–12.0)
Prior antimicrobial drugs			
Within past 60 d	16/94 (17.0)	13/359 (3.6)	4.7 (2.2–10.0)
Within past 2 d	4/27 (14.8)	25/426 (5.9)	2.5 (0.8–6.7)
IV antimicrobial drugs taken within past 30 d	4/26 (15.4)	25/425 (5.9)	2.6 (0.8–6.9)
LTC within past 90 d	1/3 (33.3)	28/450 (6.2)	5.4 (0.3–14.9)
Hospital admittance within past 90 d	5/42 (11.9)	24/411 (5.8)	2.0 (0.7–5.2)
Travel outside United States within past 90 d	4/17 (23.5)†	25/436 (5.7)	4.1 (1.3–10.1)
UTI caused by fluoroquinolone-resistant *E. coli*			
Within past year	5/9 (55.6)	24/444 (5.4)	10.3 (3.8–17.5)
Within past 90 d	5/8 (62.5)	24/445 (5.4)	11.6 (4.4–18.3)
UTI caused by ceftriaxone-resistant *E. coli*			
Within past year	5/6 (83.3)	24/447 (5.4)	15.5 (6.2 −19.2)
Within past 90 d	5/5 (100.0)	24/448 (5.4)	18.7 (8.0–18.7)
Fluoroquinolone resistance	24/53 (45.3)	5/400 (1.3)	36.2 (14.2–104.7)

*Denominators differ because factors have a different distribution among the patient population. ESBL, extended spectrum β-lactamase; IV, intravenous; LTC, residence in a long-term care facility; UTI, urinary tract infection.  †Of these 4 patients who traveled to other regions, 3 traveled to Mexico or Central America and 1 traveled to Asia.

We further characterized 26 ESBL-producing *E. coli* isolates and 1 *K. pneumoniae* isolate. Among *E. coli* isolates, PCR identified multiple ESBL types: 22 (84.6%) produced CTX-M-15 (16 [61.5%] produced only CTX-M-15; 6 [23.1%] produced CTX-M-15 and TEM-1); 2 (7.7%) produced CTX-M-27; 1 (3.8%) produced CTX-M-14; and 1 (3.8%) produced CTX-M-14 and TEM-1. The *K. pneumoniae* isolate produced SHV-1 and CTX-M-15 ESBL types. Sixteen (61.5%) of the *E. coli* isolates were clonal type O25b-ST131.

Among patients with an *E. coli* infection, 223 (49.2%) were discharged from the ED and 229 (50.6%) were admitted to the hospital; of these, 13 (5.8%) and 18 (7.9%), respectively, were treated with an antimicrobial drug that lacked in vitro activity against their infection. Of 53 patients with a fluoroquinolone-resistant and 29 with an ESBL-producing infection, 24 (45.3%) and 22 (75.0%), respectively, were initially treated with in vitro–inactive antimicrobial drugs. Among 29 patients with an ESBL infection, 9 (31.0%) were discharged from the ED; an in vitro–inactive antimicrobial drug was initially prescribed to 7 (77.8%). Among 20 (69.0%) hospitalized patients with an ESBL infection, 15 (75.0%) were initially given an in vitro–inactive antimicrobial drug; 1 of those 15 patients was given gentamicin, 13 were given cephalosporin, and 1 was given both.

## Discussion

For 2013–2014, we found that prevalence of *E. coli* fluoroquinolone resistance was >10% for patients with uncomplicated pyelonephritis at 2 of 10 sites and >20% for patients with complicated infections at 4 of 10 sentinel sites surveyed in the United States. *E. coli* fluoroquinolone resistance was particularly prevalent in groups with antimicrobial drug resistance risk. These rates exceed thresholds for the 2010 IDSA treatment guidelines, which recommend consideration of an additional antimicrobial drug of a different class and other agents (*9*). Data from a similar study we conducted during 2000–2004 (*15*) indicate that, among all healthcare-seeking ED patients with acute pyelonephritis, the prevalence of fluoroquinolone-resistant *E. coli* increased from 3.9% during 2000–2004 to 11.7% during 2013–2014. During 2000–2004, we found no infections caused by ESBL-producing bacteria. As in other parts of the world, ESBL-producing *Enterobacteriaceae* are emerging among patients with community-acquired UTI in the United States. For patients with uncomplicated and complicated pyelonephritis caused by *E. coli*, we found that 2.6% and 12.2%, respectively, had infection caused by an ESBL-producing organism; rates were even higher for patients with risk factors. The globally disseminated, multidrug-resistant clone ST131, which produces CTX-M-15 β-lactamase, accounted for 85.2% of these infections. Of ESBL-infected patients, about one third lacked traditional antimicrobial resistance risk factors (i.e., antimicrobial drug or healthcare-setting exposure or international travel), suggesting that these isolates are now endemic in some US communities. Among ESBL-infected patients, about three quarters were initially treated with an antimicrobial drug lacking in vitro activity, including the sickest patients who required hospitalization. We did not collect outcome data, but lack of in vitro activity of the antimicrobial drug used for treatment has been associated with relatively poor response rates among patients with pyelonephritis (*1**,**10**,**11*).

Previous surveys have suggested that *E. coli* fluoroquinolone resistance rates are increasing in the United States. Among outpatients seeking care at 30 US centers during 2003–2004, 59 (6.8%) of 862 *E. coli* isolates were resistant to ciprofloxacin (*2*). Another analysis of >12 million urine specimens from US outpatient centers found that the *E. coli* fluoroquinolone resistance rate increased from 3.0% in 2000 to 17.1% in 2010 (*3*). Such laboratory-based resistance surveillance data may exaggerate the prevalence of resistance because patients for whom cultures are performed would be expected to have received prior therapy and to have had healthcare exposure more frequently than patients without cultures. Isolate-driven studies require retrospective review of records, which have missing and inaccurate data; also, uncertainty may exist regarding whether a specimen is from a patient with an actual clinical infection, rather than being a colonized or contaminated specimen. In routine practice, providers typically estimate local resistance rates on the basis of an antibiogram published by the local hospital laboratory. Resistance rates determined from antibiograms are prone to bias and indicate only whether the specimen was obtained from an outpatient or inpatient location.

In contrast, we conducted syndromic surveillance of patients who sought care at a geographically diverse network of US EDs. We studied acute pyelonephritis because it is a distinct clinical syndrome for which cultures are routinely obtained and because isolates grown are less likely to be contaminants or colonizers, compared with those from patients with suspected cystitis. Historical data were obtained by real-time patient interviews, enabling accurate classification of complicated and uncomplicated pyelonephritis and ascertainment of antimicrobial drug resistance risk factors. To identify biases, we compared characteristics of enrolled and nonenrolled qualifying patients and found their characteristics to be similar, suggesting the validity of our sampling. Consequently, our data and that of other studies indicate that, in some parts of the United States, the rate of *E. coli* fluoroquinolone resistance among uropathogens is >10% among patients with uncomplicated pyelonephritis and >20% among those with complicated infections. However, variability exists; among patients with uncomplicated pyelonephritis, 3 of 10 sites had fluoroquinolone resistance rates <5%. Consistent with findings of previous investigations, we found *E. coli* fluoroquinolone resistance associated with complicated infection, prior use of antimicrobial drugs and fluoroquinolone, hospitalization, and prior UTI caused by a fluoroquinolone- or ceftriaxone-resistant organism (*16**,**17*).

By using active, prospective surveillance, we found that ESBL-producing *E. coli* infections have now emerged to a considerable degree among patients with clinically confirmed community-acquired infections in parts of the United States, including among persons lacking commonly recognized antimicrobial drug resistance risk factors. This new observation is not unexpected, given the reported epidemiology of ESBL-producing *Enterobacteriaceae* infections in communities outside North America. CTX-M enzymes are currently the most prevalent ESBL types worldwide. The ST131 clone is largely responsible for the international epidemic caused by CTX-M-15–producing *E. coli*, including infections seen in the United States (*7**,**18**,**19*).

A few US laboratory-based surveillance studies have reported community-acquired ESBL infections. Peirano et al. (*19*) described ESBL-producing *E. coli* isolates from 30 community-dwelling patients at 5 Chicago-area hospitals during 2008. These ESBL-producing strains represented 2%–8% of *E. coli* isolates at each hospital. Khawcharoenporn et al. (*20*) reported that ≈5% of *Enterobacteriaceae* isolates from ED patients with presumed UTI during 2008–2009 were ESBL-producing, although specific risk data were not provided. Doi et al. (*7*) reviewed records of patients with cultures that grew ESBL-producing *E. coli* isolates at 1 hospital in each of 5 US cities during 2009–2010. Among 13,270 *E. coli* isolates, 523 (3.9%) were ESBL producing. Of the 291 patients infected or colonized with ESBL-producing *E. coli* as outpatients, infections of 107 (36.8%) were thought to be community associated. Community ESBL-producing *E. coli* isolates were resistant to multiple agents: 87.5% to ciprofloxacin or levofloxacin and 39.4% to gentamicin. All isolates from that study were susceptible to a carbapenem; we also found that all ESBL-producing isolates in our investigation were susceptible to a carbapenem.

Studies from outside North America have identified several characteristics associated with ESBL infection, such as recurrent and complicated UTI; advanced age; recent hospitalization; use of a β-lactam or fluoroquinolone; travel to Asia, Middle East, or Africa; and fresh water swimming (*5**,**21**–**23*). Banerjee et al. (*23*) conducted a case–control study among adults with *E. coli* clinical isolates cultured in the Chicago area and found that ESBL infection was associated with travel to India, ciprofloxacin use, and age. We found ESBL-producing *E. coli* infection associated with complicated infection, prior antimicrobial drug use, travel outside North America, and prior UTI resulting from fluoroquinolone- or ceftriaxone-resistant infection. The importance of investigating past susceptibility data when considering empirical treatment is highlighted by our observation that both fluoroquinolone resistance and ESBL-production were associated with previous resistant infections.

Our study has limitations. We were unsuccessful in enrolling all consecutive patients, which may have introduced bias in our selection of patients. However, our audit of eligible case-patients showed similarity of enrolled and nonenrolled patients, including their *E. coli* antimicrobial drug susceptibility rates; furthermore, most (>97%) enrolled patients had urine cultures collected, reducing potential bias. The prevalence of ESBL-producing strains may have been underestimated because we did not use ESBL-selective media and used only ceftriaxone instead of several advanced-generation cephalosporins to screen for presence of ESBL-producing strains. However, this method enabled us to have greater site participation; furthermore, screening isolates with a ceftriaxone MIC >1 µg/mL has been reported to have a sensitivity >98% on the basis of phenotypic testing (*24*). In addition, some patients may not have had pyelonephritis if a contaminated specimen was misinterpreted as noncontaminated, although >97% of patients had urine collected by a technique that minimizes contamination (i.e., clean catch, urethral catheterization, or suprapubic aspiration), and the diagnosis of pyelonephritis was further supported by clinical assessment. Our definition of confirmed infection as growth of 1 uropathogen at >10^4^ CFU/mL may have missed some cases of pyelonephritis, although only ≈5% grew a single uropathogen at <10^4^ CFU/mL or grew >1 uropathogen at >10^4^ CFU/mL. Our hospitals were large US urban centers and may not represent patients in other settings, emphasizing the importance of local surveillance.

IDSA treatment guidelines for acute uncomplicated pyelonephritis recommend that, if the fluoroquinolone-resistance rate is >10%, then in addition to a fluoroquinolone, an agent of another class (i.e., ceftriaxone or gentamicin) should be administered (*9*). Our findings indicate that fluoroquinolone resistance rates for *E. coli* are approaching or exceed this threshold for patients with uncomplicated pyelonephritis in many parts of the United States. For uncomplicated cystitis, the guidelines recommend alternative agents if the resistance rate is >20%, which is the current situation for fluoroquinolones in many settings for patients with complicated pyelonephritis. Unfortunately, we found that only one half to two thirds of fluoroquinolone-resistant *E. coli* isolates were susceptible to ceftriaxone or gentamicin. Rates of fluoroquinolone-resistant and ESBL-producing *E. coli* infections correlate to geographic location. Prior exposure to antimicrobial drugs or a healthcare setting, travel outside the United States, and a history of an antimicrobial drug–resistant infection substantially increases the chance that a person will have a current fluoroquinolone-resistant or ESBL-producing *E. coli* infection. Therefore, in settings with high fluoroquinolone resistance rates, in settings where ESBL-producing *Enterobacteriaceae* infections have emerged, or among persons with antimicrobial drug resistance risk factors (especially patients with or at risk for severe sepsis), healthcare providers should consider empirical treatment with a carbapenem or another agent found to be consistently active on the basis of the local antibiogram. In this study, ≈50% of patients with pyelonephritis were managed as outpatients. Currently, no oral antimicrobial drugs with consistent in vitro activity are available for empirical treatment of pyelonephritis caused by ESBL-producing *E. coli* uropathogens. Our findings, including the variability in the prevalence of resistance by site, show that increased local efforts to enhance surveillance for antimicrobial drug resistance are necessary to best inform treatment decisions. Furthermore, availability of new antimicrobial drugs must be expedited.

Technical Appendix Study methods and materials and tables comparing enrolled and nonenrolled patients and *Escherichia coli* antimicrobial drug resistance rates for patients with pyelonephritis by study sites.
