# Supplementary material for: Fluoroquinolone-Resistant and Extended-Spectrum β-Lactamase–Producing Escherichia coli Infections in Patients with Pyelonephritis, United States
Source: Emerg Infect Dis. 2016 Sep;22(9):1594–603. doi: 10.3201/eid2209.160148 (PMC4994338; doi:10.3201/eid2209.160148)
Supplement: Technical Appendix — Study methods and materials and tables comparing enrolled and nonenrolled patients and Escherichia coli antimicrobial drug resistance rates for patients with pyelonephritis by study sites. [file 16-0148-Techapp-s1.pdf]

Article DOI: http://dx.doi.org/10.3201/eid2209.160148

Fluoroquinolone-Resistant and Extended-Spectrum β-Lactamase–Producing *Escherichia coli* infections in Patients with Pyelonephritis, United States

Technical Appendix

Methods and Materials

Study Sites

The study was conducted at the following emergency department sites in the United States: Olive View–University of California, Los Angeles Medical Center, Los Angeles, CA; Hennepin County Medical Center, Minneapolis, MN; University of New Mexico Health Sciences Center, Albuquerque, NM; Bellevue Hospital Center, New York, NY; Maricopa Medical Center, Phoenix, AZ; Oregon Health & Science University, Portland, OR; Temple University School of Medicine, Philadelphia, PA; University of Missouri–Kansas City, Kansas City, MO; Brigham and Women's Hospital, Boston, MA; The Johns Hopkins Hospital, Baltimore, MD.

Confirmation of Extended-Spectrum β-Lactamase Production and Molecular Characterization

Study sites referred ceftriaxone-nonsusceptible (i.e., MIC >1 µg/mL) *Enterobacteriaceae* isolates to the laboratory at International Health Management Associates, Inc. ([IHMA] Schaumburg, IL, USA) for specie identification. IHMA confirmed specie identification by matrix-assisted laser desorption ionization time of flight mass spectrometry (Bruker Daltronics, Bremen, Germany). Temple University School of Medicine referred all *Escherichia coli* isolates. MICs to ceftriaxone were determined by broth microdilution by using custom-manufactured dehydrated panels (MicroScan, Siemens Medical Solutions Diagnostics, West Sacramento, CA, USA), following Clinical and Laboratory Standards Institute guidelines (*1*).

All *E. coli*, *Klebsiella pneumoniae*, and *Proteus mirabilis* isolates with ceftriaxone MIC values >1 µg/mL were further screened at IHMA for extended-spectrum β-lactamase (ESBL) activity by using a disk diffusion assay testing cefotaxime (30 µg), cefotaxime/clavulanic acid (30/10 µg), ceftazidime (30 µg), and ceftazidime/clavulanic acid (30/10 µg) disks (BBL Sensi-Disc, Becton Dickinson, Franklin Lakes, NJ, USA). An isolate was confirmed as phenotypically ESBL positive if the inhibition zone diameter of the combination disk increased ≥5 mm, compared with that of only cephalosporin (*1*).

All isolates subjected to the phenotypic ESBL test were screened for the presence of β-lactamase genes encoding ESBLs (TEM-, SHV-, CTX-M–, VEB-, PER-, GES-type), original-spectrum β-lactamases (TEM-1, SHV-1, SHV-11), plasmid-mediated AmpC β-lactamases (ACC-, ACT-, CMY-, DHA-, FOX-, MIR-, MOX-type), and carbapenemases (KPC-, OXA-48-, IMP-, VIM-, NDM-, SPM-type) by using previously described multiplex PCR assays (*2*). Enzyme variants were identified by DNA sequencing and comparison with sequences in public databases maintained by the National Center for Biotechnology Information (www.ncbi.nlm.nih.gov) and the Lahey Clinic (www.lahey.org/studies). *E. coli* isolates with ceftriaxone MIC values >1 µg/mL were screened by PCR for the presence of sequence type 131–associated single-nucleotide polymorphisms in *mdh* and *gyr*B (*3*) and were screened separately for O25b- and O16-specific *rfb* variants (*4*). Sequence type 131 control strains were generously provided by D. Hoban and K. Nichol at the University of Manitoba (Winnipeg, Manitoba, Canada). Three *E. coli* isolates identified as ESBL producers at the Temple University site were not saved and sent to the IHMA laboratory but were confirmed to be ESBL by the Phoenix ESBL test (Becton Dickenson).

Audit Comparing Enrolled and Nonenrolled Cases

All sites conducted an audit to compare characteristics of enrolled and nonenrolled eligible patients. Site investigators screened emergency department (ED) patient logs to find patients with ED discharge of pyelonephritis (i.e., International Classification of Diseases, Ninth Revision, code 590.8 [pyelonephritis, unspecified]) and a documented temperature $\geq$38.0^{o}C; they then reviewed medical records for these patients. Patients meeting study eligibility criteria had the following data collected: age, sex, ED disposition (i.e., discharged home, admitted to ward or ICU, admitted to ED observation, or left ED against medical advice), proportion pathogen and *E.*

coli growth, and *E. coli* susceptibility to trimethoprim/sulfamethoxazole, ceftriaxone, and fluoroquinolones.

References

1. Clinical and Laboratory Standards Institute. Performance standards for antimicrobial susceptibility testing; twenty-fifth informational supplement. CLSI document M100-S25. Wayne (PA): Clinical and Laboratory Standards Institute; Jan 2015.

2. Lob SH, Kazmierczak KM, Badal RE, Hackel MA, Bouchillon SK, Biedenbach DJ, et al. Trends in susceptibility of *Escherichia coli* from intra-abdominal infections to ertapenem and comparators in the United States according to data from the SMART program, 2009 to 2013. Antimicrob Agents Chemother. 2015;59:3606–10. http://dx.doi.org/10.1128/AAC.05186-14

3. Johnson JR, Menard M, Johnston B, Kuskowski MA, Nichol K, Zhanel GG. Epidemic clonal groups of *Escherichia coli* as a cause of antimicrobial-resistant urinary tract infections in Canada, 2002 to 2004. Antimicrob Agents Chemother. 2009;53:2733–9. http://dx.doi.org/10.1128/AAC.00297-09

4. Johnson JR, Clermont O, Johnston B, Clabots C, Tchesnokova V, Sokurenko E, et al. Rapid and specific detection, molecular epidemiology, and experimental virulence of the O16 subgroup within *Escherichia coli* sequence type 131. J Clin Microbiol. 2014;52:1358–65. http://dx.doi.org/10.1128/JCM.03502-13

Technical Appendix Table 1. Comparison of eligible enrolled and nonenrolled US emergency department patients, July 2013–December 2014*

Characteristic	Enrolled, no. (%) N = 817	Nonenrolled, no. (%) N = 414
Median age (range), y	37 (18–89)	37 (18–79)
Sex		
F	684 (83.7)	335/408 (82.1)
M	133 (16.3)	73/408 (17.9)
Hospitalized	478 (58.5)	200/411 (48.7)
Pathogen growth in urine culture	644 (78.8)	336 (81.2)
E. coli growth in urine culture	480 (58.8)	238 (57.4)
E. coli susceptible to TMP/SMX	308 (64.2)	164/248 (66.1)
E. coli susceptible to ceftriaxone	442/480 (92.1)	181/191 (94.8)
E. coli susceptible to levofloxacin	303/339 (89.4)	113/121 (93.4)
E. coli susceptible to ciprofloxacin	369/424 (87.0)	152/172 (88.4)

* Denominators are indicated if data were missing. TMP/SMX, trimethoprim-sulfamethoxazole.

Technical Appendix Table 2. *Escherichia coli* antimicrobial drug resistance rates among 272 US emergency department patients with uncomplicated pyelonephritis by study site, July 2013–December 2014

	Antimicrobial drug resistance, no./total tested (%)										
Site	Ampic	TMP/SMX	Genta	Cefaz	Ceftr	Cipro	Levof	Imipe	Ertap	Merop	Dorip
All sites	152/272 (55.9)	111/272 (40.8)	19/261 (7.3)	18/219 (8.2)	7/272 (2.6)	15/237 (6.3)	10/195 (5.1)	0/90 (0)	0/111 (0)	0/96 (0)	0/74 (0)
New York, NY	17/24 (70.8)	13/24 (54.2)	3/24 (12.5)	4/24 (16.7)	2/24 (8.3)	3/24 (12.5)	NA	0/24 (0)	NA	0/23 (0)	NA
Boston, MA	7/16 (43.8)	4/16 (25.0)	1/9 (11.1)	1/2 (50.0)	0/16 (0)	0/16 (0)	0/10 (0)	0/1 (0)	0/5 (0)	0/3 (0)	NA
Minneapolis, MN	7/21 (33.3)	5/21 (23.8)	1/21 (4.8)	NA	1/21 (4.8)	0/2 (0)	1/21 (4.8)	N/A	0/21 (0)	0/19 (0)	0/2 (0)
Baltimore, MD	6/13 (46.2)	5/13 (38.5)	0/13 (0)	1/13 (7.7)	0/13 (0)	3/13 (23.1)	NA	0/3 (0)	0/13 (0)	0/13 (0)	NA
Phoenix, AZ	16/22 (72.7)	12/22 (54.5)	0/22 (0)	0/4 (0)	0/22 (0)	1/22 (4.5)	1/22 (4.5)	NA	NA	NA	NA
Portland, OR	5/8 (62.5)	4/8 (50.0)	1/8 (12.5)	1/8 (12.5)	0/8 (0)	0/8 (0)	NA	NA	NA	NA	NA
Los Angeles, CA	44/71 (62.0)	32/71 (45.1)	9/71 (12.7)	5/71 (7.0)	3/71 (4.2)	4/71 (5.6)	4/71 (5.6)	N/A	0/71 (0)	NA	0/71 (0)
Philadelphia, PA	28/53 (52.8)	18/53 (34.0)	1/53 (1.9)	4/53 (7.5)	1/53 (1.9)	3/ 53 (5.7)	3/52 (5.8)	0/53 (0)	0/1 (0)	0/1 (0)	0/1 (0)
Kansas City, MO	9/19 (47.4)	8/19 (42.1)	1/19 (5.3)	1/19 (5.3)	0/19 (0)	0/3 (0)	1/19 (5.3)	N/A	NA	0/18 (0)	NA
Albuquerque, NM	13/25 (52.0)	10/25 (40.0)	2/21 (9.5)	1/25 (4.0)	0/25 (0)	1/25 (4.0)	NA	0/9 (18.2)	NA	0/19 (0)	NA

*Ampic, ampicillin; Cefaz, cefazolin; Ceftr, ceftriaxone; Cipro, ciprofloxacin; Dorip, doripenem; Ertap, ertapenem; Genta, gentamicin; Imipe, imipenem; Levof, levofloxacin; Merop, meropenem; NA, none tested; TMP/SMX, trimethoprim-sulfamethoxazole.

Technical Appendix Table 3. *Escherichia coli* antimicrobial-drug resistance rates among 181 US emergency department patients with complicated pyelonephritis by study site, July 2013–December 2014

	Antimicrobial drug resistance, no./total tested (%)										
Site	Ampic	TMP/SMX	Genta	Cefaz	Ceftr	Cipro	Levof	Imipe	Ertap	Merop	Dorip
All sites	107/181 (59.1)	54/181 (29.8)	24/175 (13.7)	34/148 (23.0)	28/181 (15.5)	33/160 (20.6)	23/130 (17.7)	0/45 (0)	0/90 (0)	0/65 (0)	0/65 (0)
New York, NY	12/21 (57.1)	4/21 (19.0)	4/21 (19.0)	8/21 (38.1)	4/21 (19.0)	7/21 (33.3)	1/1 (100.0)	0/21 (0)	NA	0/21 (0)	NA
Boston, MA	4/6 (66.7)	2/6 (33.3)	0/3 (0)	0/1 (0)	0/6 (0)	0/6 (0)	0/3 (0)	NA	NA	NA	NA
Minneapolis, MN	6/13 (46.2)	4/13 (30.8)	2/12 (16.7)	1/1 (100.0)	1/13 (7.7)	0/1 (0)	2/13 (15.4)	NA	0/12 (0)	0/12 (0)	0/1 (0)
Baltimore, MD	7/10 (70.0)	4/10 (40.0)	2/10 (20.0)	1/10 (10.0)	1/10 (10.0)	2/10 (20.0)	NA	0/4 (0)	0/10 (0)	0/10 (0)	NA
Phoenix, AZ	12/21 (57.1)	11/21 (52.4)	1/21 (4.8)	1/5 (20.0)	3/21 (14.3)	3/21 (14.3)	3/21 (14.3)	NA	0/1 (0)	0/1 (0)	NA
Portland, OR	6/8 (75.0)	2/8 (25.0)	2/8 (25.0)	4/8 (50.0)	2/8 (25.0)	4/8 (50.0)	0/1 (0)	NA	0/3 (0)	0/3 (0)	NA
Los Angeles, CA	45/64 (70.3)	19/64 (29.7)	9/64 (14.1)	15/64 (23.4)	13/64 (20.3)	13/64 (20.3)	13/64 (20.3)	NA	0/64 (0)	NA	0/64 (0)
Philadelphia, PA	8/18 (44.4)	4/18 (22.2)	2/18 (11.1)	3/18 (16.7)	3/18 (16.7)	3/18 (16.7)	3/18 (16.7)	0/18 (0)	NA	NA	NA
Kansas City, MO	3/9 (33.3)	2/9 (22.2)	1/9 (11.1)	0/9 (0)	0/9 (0)	NA	1/9 (11.1)	NA	NA	0/9 (0)	NA
Albuquerque, NM	4/11 (36.4)	2/11 (18.2)	1/9 (11.1)	1/11 (9.1)	1/11 (9.1)	1/11 (9.1)	NA	0/2 (0)	NA	0/9 (0)	NA

*Ampic, ampicillin; Cefaz, cefazolin; Ceftr, ceftriaxone; Cipro, ciprofloxacin; Dorip, doripenem; Ertap, ertapenem; Genta, gentamicin; Imipe, imipenem; Levof, levofloxacin; Merop, meropenem; NA, none tested; TMP/SMX, trimethoprim-sulfamethoxazole
